# Cellular translocation and secretion of sialidases

**DOI:** 10.1016/j.jbc.2024.107671

**Published:** 2024-08-14

**Authors:** Majdi A. Aljohani, Hiroaki Sasaki, Xue-Long Sun

**Affiliations:** 1 Department of Chemistry, Chemical and Biomedical Engineering and Center for Gene Regulation in Health and Disease (GRHD), Cleveland State University, Cleveland, Ohio, USA; 2 Faculty of Applied Medical Sciences, Department of Medical Laboratory Technology, University of Tabuk, Tabuk, Saudi Arabia; 3 Department of Pharmacognosy and Phytochemistry, Meiji Pharmaceutical University, Kiyose-shi, Tokyo, Japan

**Keywords:** sialic acid, sialidase, sialylation, desialylation, exosome

## Abstract

Sialidases (or neuraminidases) catalyze the hydrolysis of sialic acid (Sia)-containing molecules, mostly the removal of the terminal Sia on glycans (desialylation) of either glycoproteins or glycolipids. Therefore, sialidases can modulate the functionality of the target glycoprotein or glycolipid and are involved in various biological pathways in health and disease. In mammalian cells, there are four kinds of sialidase, which are Neu1, Neu2, Neu3, and Neu4, based on their subcellular locations and substrate specificities. Neu1 is the lysosomal sialidase, Neu2 is the cytosolic sialidase, Neu3 is the plasma membrane-associated sialidase, and Neu4 is found in the lysosome, mitochondria, and endoplasmic reticulum. In addition to specific subcellular locations, sialidases can translocate to different subcellular localizations within particular cell conditions and stimuli, thereby participating in different cellular functions depending on their loci. Lysosomal sialidase Neu1 can translocate to the cell surface upon cell activation in several cell types, including immune cells, platelets, endothelial cells, and epithelial cells, where it desialylates receptors and thus impacts receptor activation and signaling. On the other hand, cells secrete sialidases upon activation. Secreted sialidases can serve as extracellular sialidases and cause the desialylation of both extracellular glycoproteins or glycolipids and cell surface glycoproteins or glycolipids on their own and other cells, thus playing roles in various biological pathways as well. This review discusses the recent advances and understanding of sialidase translocation in different cells and secretion from different cells under different conditions and their involvement in physiological and pathological pathways.

### Sialic acids, sialylation, and desialylation

Sialic acids (Sias) are a family of 9-carbon-containing acidic monosaccharides that are attached to either the galactose (Gal), or *N*-acetyl galactosamine (GalNAc) unit *via* α2,3- or α2,6-linkage, or to another Sia *via* α2,8-linkage at the terminus of the glycan structures of glycoproteins and glycolipids (Fig. 1) (1, 2). Due to their terminal location, hydrophilic and negative properties, Sias have various functions, such as (i) exerting physicochemical effects on the glycoconjugates, (ii) serving as recognition components, or (iii) masking the binding sites of the glycoconjugates and thus are involved in various biological pathways (1, 2, 3, 4). The levels and linkages of Sias on the glycoproteins and glycolipids (sialylation) are controlled by specific sialyltransferases. In addition, the removal of Sias from them (desialylation) is catalyzed by sialidases under various biological pathways (3, 4). Desialylation leads to reducing a negative charge and changing the conformation and accessibility of the glycoconjugate and thus is able to modulate its functionality and stability in a biological pathway (5, 6). Therefore, sialidases play important roles in many physiological and pathological processes, including cellular communication, immunological response, cell adhesion and migration, *etc.*

**Figure 1:**
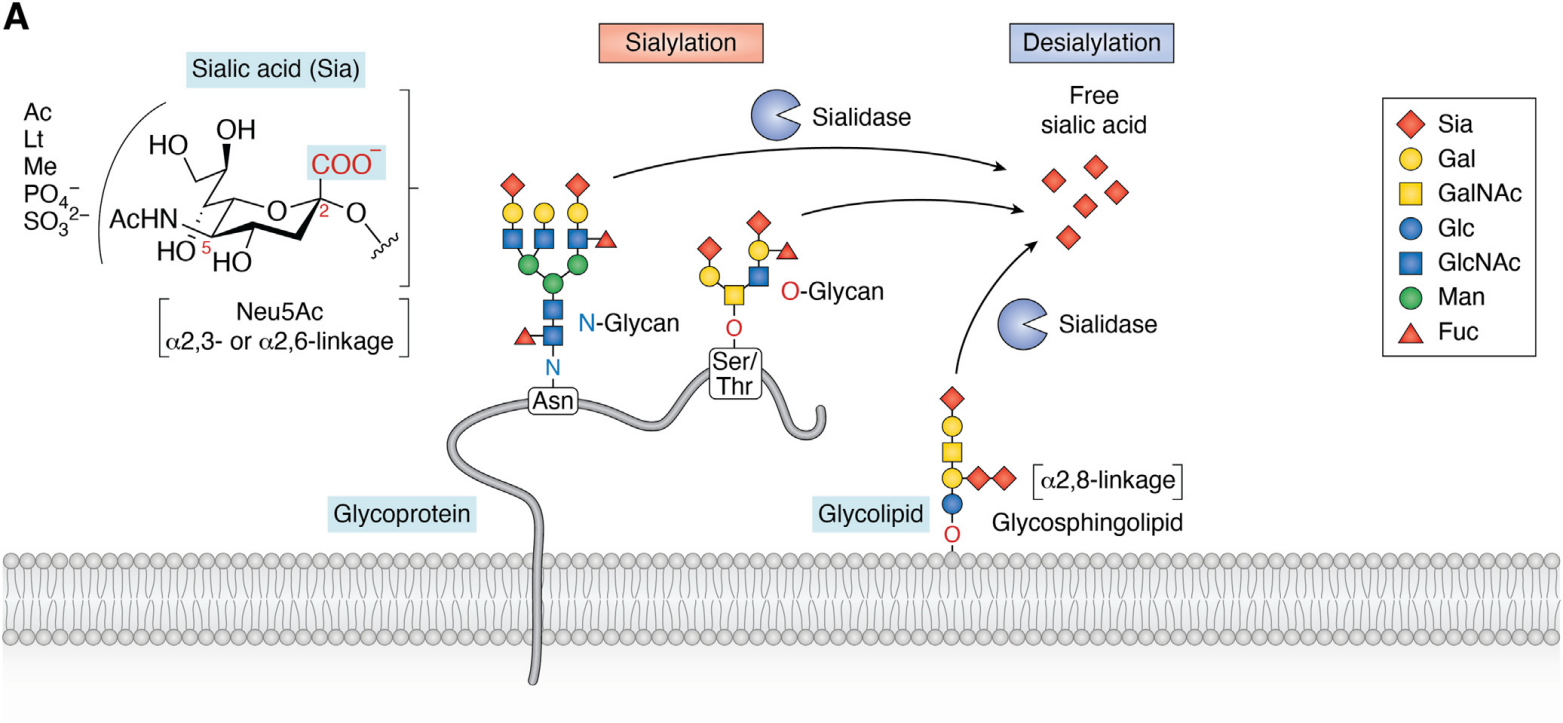
**Sialic acids (Sias), sialylation, and desialylation of cell surface glycoprotein and glycolipid by sialidase**.

### Sialidase expression and cellular distribution

Sialidases (or Neuraminidases) are glycosidases responsible for the desialylation of glycoconjugates on the cell surface as well as inside the cells under various conditions. Mammalian sialidases have been classified into four types (Neu1, Neu2, Neu3, and Neu4) according to their subcellular localizations and substrate specificities (7, 8). Each one of the four sialidases is encoded by a unique gene (9). Neu1 is typically located in the lysosome, Neu2 is in the cytosol, and Neu3 is associated with plasma membranes, while Neu4 is found in the lysosome, mitochondria, and endoplasmic reticulum (ER) as well, suggesting different biological functions of each sialidase (Fig. 2) (7, 8, 10, 11).

**Figure 2:**
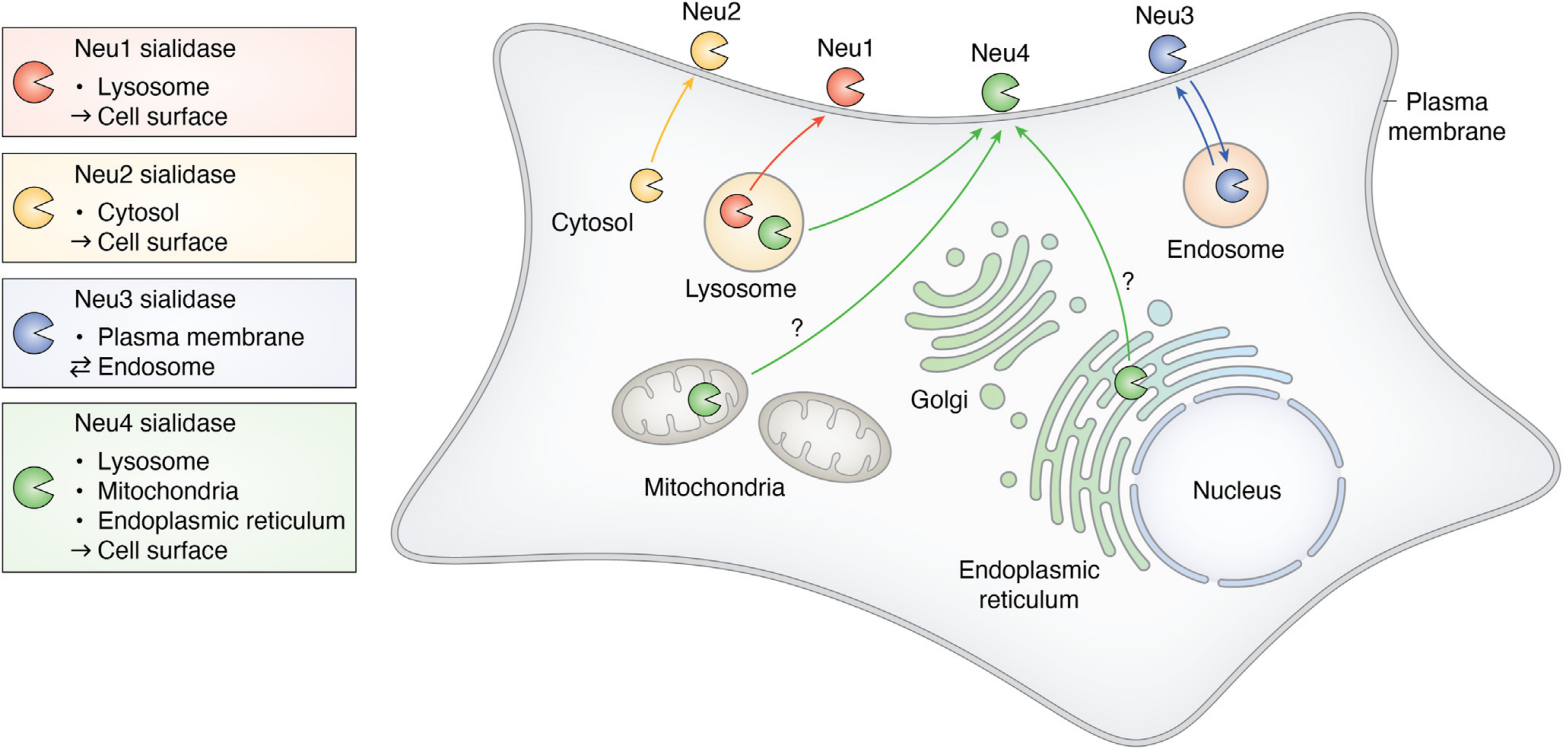
**Subcellular distribution of sialidases and their translocations.** Neu1 is typically located in the lysosome and can translocate to the cell surface, Neu2 in cytosol can translocate to the cell surface, Neu3 on the plasma membranes can translocate to the endosome, while Neu4 is located in lysosome, mitochondria, and endoplasmic reticulum and can translocate to the cell surface as well.

Neu1 is synthesized at the rough ER and glycosylated in the ER/Golgi before being transferred into the lysosome. Neu1 exists in a complex with protective protein/cathepsin A (PPCA) and β-galactosidase in the lysosome, where it activates and protects Neu1 against proteolytic degradation by lysosomal peptidases (10). The main function of Neu1 is to degrade internalized or endocytosed sialylglycans, which is required for normal cellular functions. Malfunctions and mutations of Neu1 are linked to a variety of pathological disorders (7). It predominantly cleaves α2,3- and, to a lesser degree, α2,6- and α2,8-linked Sias from glycoproteins and glycolipids (7). As a cytosolic sialidase, Neu2 desialylates cytosolic sialylglycans at neutral pH and has similar substrate and linkage specificity to Neu1 (7, 12). Neu3 is a plasma membrane-bound sialidase, where it cleaves Sias from gangliosides (7), which may be mediated by a binding domain for a hydrophobic aglycone, for example an alkyl chain of a lipid (11). Neu4 is first identified as a lysosomal sialidase (13) and is found in mitochondria and the ER later. In fact, there are two forms found for Neu4, a long Neu4 and a short Neu4, differing in the presence of a 12-amino-acid sequence at the N-terminus (14). The long Neu4 form localizes in mitochondria, while the short Neu4 form is associated with the ER.

The expression levels of the four sialidases are quite different. Neu1 shows 10 to 20 times higher expression than Neu3 and Neu4, and ∼10^3^-10^2^ higher expression than Neu2 (15). Neu1 is highly expressed in the kidney, pancreas, skeletal muscle, liver, lungs, placenta and brain (16). Neu2 is generally expressed at higher levels in the placenta and the testis (17). Neu3 shows higher expression in the adrenal gland, skeletal muscle, heart, testis, and thymus (18), while Neu4 is highly expressed in the brain, skeletal muscle, heart, placenta and liver (13). Structurally, Neu1 shares only 18 to 24% sequence identity with the other three sialidases, whereas Neu2-4 share 34 to 40% identity amongst themselves (15).

In addition to specific subcellular locations, mammalian sialidases could translocate to another subcellular location upon cell activation (Table 1, Table 2, Table 3, Table 4, Table 5), where they cleave Sia from the target glycoconjugate and activate a specific signaling pathway (Fig. 2). On the other hand, sialidase activities were detected in the cell culture medium, indicating the secretion of sialidases from the cells upon activation, including platelets re-warmed from refrigeration (19, 20, 21). Secreted sialidases could serve as extracellular sialidases (21) and cause the desialylation of both extracellular glycoproteins and glycolipids and cell surface glycoproteins and glycolipids on their own and other cells. Several excellent reviews have been published on the physiological function of sialidases and desialylation (5, 6, 7, 8, 9, 10). This review discusses the translocation and secretion of sialidases in different cells and their involvement in different biological pathways.

**Table 1:** Neu1 translocation in immune cells

Cell lines	Stimulus	Target	Diseases	Ref.
THP-1 monocytes	PMA	MHCII and lamp-2	N/A	(40)
	IL-1β and LPS	TLR4	Atherosclerosis	(41)
	Elastin-derived peptides (EDP)	CD36	Atherosclerosis	(35)
Murine monocytes	LPS	TLR4	N/A	(33, 34)
BMA macrophages	Nerve growth factor (NGF)	TrkA	N/A	(39)
Murine Macrophages	BAPN	Fc receptors for IgG	Aortic dissection (AD)	(36)
J774A.1 cells	Leishmania donovani	TLR4	Leishmaniasis	(31)
Ra2 microglia cells	LPS	Cell surface sialic acid	N/A	(37)
Murine DCs	LPS	TLR4	N/A	(33)
Murine Neutrophils	LPS	TLR4	N/A	(32, 33)
Human Neutrophils	PMA	Beta integrin (CD11b/CD18)	N/A	(38)
Human T lymphocyte	Anti-CD3 and anti-CD28 IgG	CD4 and CD8	N/A	(28)

**Table 2:** Neu1 translocation in platelets

Cell lines	Stimulus	Target	Diseases	Ref.
Human platelets	Anti-GPIb/IX	Cell surface Sia	Immune thrombocytopenia (ITP)	(46)
	Ristocetin, ADP VWF, fibrinogen	αIIbβ3 -integrin	N/A	(44)
	Allo-HSCT	GPIbα	Prolonged isolated thrombocytopenia (PT)	(49)
	Snake venoms	Cell surface Sia	Coagulopathy-independent thrombocytopenia	(48, 55)
	Anti-GPIIb/IIIa	Cell surface Sia	Immune thrombocytopenia (ITP)	(48)
	VWF		Dengue-associated thrombocytopenia	(45)
Murine platelets	Refrigeration	GPIbα	N/A	(19)
	CD8+ T cells	Cell surface Sia	Immune thrombocytopenia (ITP) Prolonged isolated thrombocytopenia (PT)	(19, 54)
	IgG	αiibβ3	Acquired Glanzmann thrombasthenia	(53)
	VWF	αIIb and β3	Type 2B von Willebrand disease	(47)

**Table 3:** Neu1 translocation in epithelial cells

Cell lines	Stimulus	Target	Diseases	Ref.
A549 human small airway epithelial cells	TGF-β1 (10 ng/ml)	Epithelial cell surface sialic acid	Pulmonary fibrosis	(56)
	Pa flagellin	EGFR and MUC1	N/A	(60, 61)
HepG2 cells	Ambroxol	Insulin receptor	T2DM	(58)

**Table 4:** Neu1 translocation in endothelial cells

Cell lines	Stimulus	Target	Diseases	Ref.
HPMECs, HPAECs	Over expression	CD31	N/A	(62, 65)
HPMVECs	Over expression	EC surface Sia	N/A	(64)
HPMECs	DENV2, WNV NS1	TLR4, CD31	Dengue infection	(62, 65)

**Table 5:** Neu1 translocation in other cells

Cell lines	Stimulus	Target	Diseases	Ref.
Aortic smooth muscle cells (AoSMCs) and fibroblasts	Antibody blocks Neu1 activity	PDGF and IGF-1 receptors	N/A	(66)
COS-7 cells	Point mutations	Dimerization of NEU1 TM2	N/A	(24)
3T3-L1 cells	Elastin degradation products (EDPs)	Insulin receptor	Insulin resistance	(67)
3T3-L1 adipocytes	LPS	MCP-1	N/A	(68)
Cortical neurons and TrkA-PC12 cells	NGF	TrkA	Neurogenesis	(39)

## Cellular translocation of sialidases

Lysosomal Neu1 could translocate towards the cell surface of various cells upon activation and stimulation, such as immune cells and platelets activation. Cytosolic Neu2 could translocate towards the plasma membrane upon cell activation and injury, such as during platelet activation. Membrane Neu3 could translocate within the plasma membrane in response to different stimuli, including the activation and differentiation of immune cells. In addition, Neu4 could translocate towards the cell surface under certain conditions. This section discusses the cellular translocation of Neu1, Neu2, Neu3, and Neu4 sialidase in different cells and in different physiological and pathological pathways.

### Neu1 sialidase translocation

Neu1 is typically found in the lysosomes, along the lysosomal membrane and lumen. Regarding the interaction of Neu1 with the membrane, studies so far have shown different results. One study suggested that Neu1 is an integral membrane protein with a C-terminal transmembrane domain and a cytosolic lysosomal targeting signal (22). However, a structural model study suggested that Neu1 associates with the membrane by lipid anchors or binds to a membrane protein (23). Interestingly, another report proposed that Neu1 contains two potential transmembrane domains and forms a dimer (24). Several functions have been confirmed for Neu1 within the lysosome, including regulation of lipid storage in lysosomes and maintenance of lysosomal pH, which facilitates protein degradation and regulates autophagy (25, 26).

Neu1 translocation to the cell surface has been observed in a variety of cells, including immune cells, platelets, endothelial cells, and epithelial cells. This was confirmed through various techniques, such as immunofluorescence, flow cytometry, immunoprecipitation, and western blotting. Some studies confirmed that Neu1 translocates to the cell surface in a complex with PPCA for proper sialidase activity (27, 28, 29), while others identified it without PPCA (24). The translocation mechanism of Neu1 is still incomplete. Lysosomal exocytosis and tyrosine phosphorylation in response to various stimuli are proposed for Neu1 translocation to the cell surface (22, 27, 30, 31). This section discusses Neu1 translocation in different cells under different cellular conditions in physiological and pathological pathways.

#### Neu1 translocation in immune cells

Cell surface sialylation and desialylation influence the functional capacity of cells on the immune system. The role of sialidase(s) and the consequent desialylation of cell surface glycoconjugates in the activation of immune cells have been investigated intensively. Lysosomal Neu1 is translocated to the cell surface of immune cells in response to different stimuli, where it desialylates specific receptors and regulates cell-cell interactions and signaling in the immune response (Table 1). Toll-like receptor 4 (TLR4) serves as a receptor for different pathogens, such as bacteria and parasites, which leads to an increase of cell surface Neu1 on dendritic cells (DCs), macrophages, and monocytes. Cell surface Neu1 removes Sia residues from the TLR4 receptor, thus eliminating steric hindrance that prevents the receptor from physically interacting with other molecules and its dimerization (31, 32, 33, 34). It is known that activation of TLR4 triggers the activation of MMP-9, which in turn promotes the translocation of Neu1 to the cell surface. A study with DCs showed that the translocation of Neu1 induced by bacterial infection leads to a positive feedback loop that decreases the inhibitory effect of sialic-acid-binding immunoglobulin-like lectins (Siglec) receptors on TLR4 signaling. Siglecs interact with Sia on TLR4 and slows down its activation. However, Neu1 translocated to the cell surface removes the Sias from TLR4, which disrupts the interaction between the TLR4 and Siglecs and thus enhances TLR4 activation, thereby increasing cytokines production (32). In terms of parasitic infection, a study found that Neu1 translocates to the cell surface of macrophages infected with *L. donovani*, where it cleaves α2,3-linked Sia of TLR4 (32). Another study showed that exposure of TLR4 galactose after desialylation is critical for the formation of the MyD88/TLR4 complex and the subsequent activation of NF-κB in response to lipopolysaccharide (LPS) stimulation. They confirmed that Neu1 activity in LPS-stimulated primary macrophage cells, macrophage and DC cell lines is responsible for the Sia removal (33). Neu1 translocation is also involved in the differentiation of monocytes into mature DCs, which are crucial immune cells responsible for antigen presentation and the initiation of an immune response (34). Changes in the glycosylation patterns of the TLR4 complex of DCs by Neu1 in response to LPS treatment are believed to have the potential to influence receptor dimerization, complex formation, and intracellular signaling (34).

In addition to TLR4, desialylation of CD36 was confirmed due to cell surface translocation of Neu1, which was recognized as an unexpected interaction partner of Neu1 (35). CD36 is a cell surface receptor essential for many functions, such as lipid metabolism, inflammation response, and fatty acid uptake by cells. They confirmed the interaction through colocalization studies and FRET (fluorescence resonance energy transfer) imaging, which showed that Neu1 and CD36 are in close proximity at the plasma membrane. Furthermore, they found that the sialylation level of CD36 is regulated by elastin-derived peptides (EDP) *via* Neu1, therefore impacting the accumulation of oxidized LDL by human macrophages (35). Moreover, another study discovered that the mRNA and protein levels of Neu1 were significantly higher in aortic dissecting tissues than in normal controls (36). They used an aortic dissecting tissue model induced by β-aminopropionitrile (BAPN) and found that BAPN reduces dissecting aneurysm formation and elastic fiber degradation since it induces Neu1 expression (36). Another study focused on the presence of polySias on the surface of microglial cells and illustrated its turnover under various conditions (37). They found that Neu1 is accumulated in a restricted region on the cell surface of Ra2 microglia immediately after LPS stimulation, leading to hydrolysis of cell surface polySia (37). PolySia has been shown to bind to brain-derived neurotrophic factor, and the degradation of polySia by Neu1 is linked to the release of brain-derived neurotrophic factor (BDNF), which is involved in various brain functions.

The β2-integrin CD11b/CD18 plays an important role in controlling neutrophil functions, including adhesion, migration, and inflammatory responses. A recent study demonstrated that desialylation of CD11b/CD18 is required for neutrophil intestinal trafficking *in vitro* and *in vivo* (38). They proposed that sialidase-catalyzed desialylation reduces conformational activation of CD11b/CD18, leading to decreased spleen tyrosine kinase (Syk) signaling. Further, inhibition of sialidase activity reduced human and murine neutrophil degranulation and superoxide release. These findings suggest that desialylation of CD11b/CD18 that reduces uncontrolled neutrophil infiltration and bystander tissue damage can be a therapeutic target for the treatment of pathologic inflammation in mucosal inflammatory disorders.

Macrophages express TrkA receptors ((39) Primary macrophage cells stimulated with nerve growth factor (NGF) induced cell surface Neu1 sialidase activity within just 1 min, which desialylates TrkA and causes TrkA receptor activation (39). Furthermore, this NGF-induced sialidase activity in live primary macrophage cells was blocked by Tamiflu, indicating Neu1’s involvement in TrkA receptor signaling (39).

Upon activation of T-lymphocytes by ant-CD3 and anti-CD28 IgG, for instance, Neu1 increases significantly on the cell surface, thereby modulating its activation and proliferation (28). Anti-CD3 and anti-CD28 IgG target specific lymphocyte cell surface receptors, mimicking the natural immune response. Neu1 was found to be closely associated with a subunit PPCA on the cell surface. This was confirmed by comparing freshly isolated lymphocytes with activated ones and applying sialidase inhibitors and galactose-binding lectin, all of which result in an increase in Neu1 on the cell surface and an increase in galactose exposure (28).

In addition to activation, during the differentiation of monocytes and the monocytic cell line, THP-1, into macrophages, lysosomal Neu1 exhibits an increase in activity, expression and translocation to the cell surface, while other cellular sialidases Neu2, Neu3, and Neu4 remain unchanged or are down-regulated (40). Also, PPCA translocates to the cell surface similarly to Neu1, suggesting the presence of linked trafficking mechanisms, which could involve in fusion of the lysosome and plasma membranes. Additionally, they demonstrated in the same study that antibody treatment or siRNA-mediated knockdown of Neu1 expression significantly affects macrophage functions, including cytokine production and bacterial ingestion. In another study, THP-1 monocytes were stimulated using a range of factors that are linked to inflammation and atherosclerosis, such as IL-1 and LPS, all which led to increased Neu1 expression and cell surface translocation (41). Further, Neu1 is involved in macrophage polarization as well, as indicated by the finding that Neu1 knockdown decreases pro-inflammatory cytokine expression (41).

Overall, cell surface translocation of Neu1 occurs in immune cells including monocytes, macrophages, dendritic cells, and T-lymphocytes (Table 1), where it removes Sia residues of cell surface receptors and modulates cell differentiation, activation, proliferation, and cytokine production as well, indicating Neu1’s regulatory role in the immune response and that Neu1 may serve as a molecular target for immunomodulation.

#### Neu1 translocation in platelets

Sialylation affects platelet lifespan and platelet activation leads to desialylation of glycans and accelerated clearance of platelets under pathological conditions (42). Desialylation of platelets has been demonstrated to be a main reason for thrombocytopenia, a complication in several infections and immune disorders. It is known that bacterial or viral infections, storage, senescence, various mutations, platelet autoantibodies, hemostasis and shear stress cause platelet desialylation (43). The translocation of Neu1 to the platelets cell surface is involved in the activation and aggregation of platelets (44). Neu1 on the platelet surface modulates the platelets’ response to various stimuli, such as von Willebrand factor, anti-glycoprotein Ib alpha (anti-GPIb), and fibrinogen, causing platelets activation and aggregation and related diseases (Table 2) (44, 45, 46, 47).

Glycoprotein Ib (GPIb) carries numerous *O*- and *N*-linked glycans on the platelet surface and plays an important role in hemostasis and thrombosis. It regulates platelet adhesion and aggregation through the GPIb-V-IX complex as a result of binding to the von Willebrand Factor (VWF) and GPIb clustering (44). Platelet membrane desialylation was observed after GPIb clustering and VWF binding due to Neu1 translocation to the platelet surface (44). In dengue patients, the binding of VWF to platelets is elevated, which triggers the translocation of Neu1 to platelet surface and increases their susceptibility to clearance (45). It was demonstrated that the neuraminidase inhibitor oseltamivir may be useful for dengue fever with severe thrombocytopenia (45). In most cases, autoantibodies against platelet GPIIbIIIa and/or the GPIb complex are the reason for the bleeding disorder known as immune thrombocytopenia (ITP) (48). Specifically, anti-GPIbα leads to significant platelet activation as a result of interaction with GPIbα on the surface of the platelets, leading to Neu1 translocation to the platelet surface (46). As a result, desialylated platelets become more susceptible to phagocytic clearance by hepatocytes and subsequent sequestration and clearance by the liver, leading to thrombocytopenia. It was shown that pan sialidase inhibitor 2-deoxy-2,3-didehydro-*N*-acetylneuraminic acid (DANA) significantly reduces this clearance, which could lead to an improvement in thrombocytopenia (46).

In prolonged isolated thrombocytopenia (PT), Neu1 and its translocation play a role in allogeneic hematopoietic stem cell transplantation (HSCT) (49). It was found that a decrease in platelet counts was linked to platelet desialylation, which was associated with increased apoptosis and phagocytosis of platelets in allogeneic HSCT patients. The increased connection between 14-3-3γ, which participates in the regulation of apoptosis, and GPIbα proteins in the platelets of patients with PT suggests a correlation between GPIbα desialylation and the onset of apoptosis. The neuraminidase inhibitor oseltamivir was proposed to manage thrombocytopenia by reducing platelet phagocytosis in allogeneic HSCT patients. Therefore, Neu1 levels in the blood and bone marrow of patients prior to transplantation could be used as a biomarker of transplant success (49).

Many studies have utilized ITP serum, which contains antibodies that target the platelet glycoprotein GPIb/IX (48) complex or GPIIb/IIIa, to treat platelets (48, 50). Surprisingly, they observed an increase in Neu1 translocation to the platelet membrane (48, 50). In addition to GPIb/IX, FcγR (Fc gamma receptor) is also implicated in apoptosis. This finding indicates that FcγRIIA participates in the platelet death pathway mediated by antibodies, particularly in the presence of anti-GPIIb/IIIa antibodies. Desialylation was observed in sera positive for antiphospholipid antibodies (APA), as confirmed by RCA-1 binding. This effect can be linked to the IgG fraction of ITP sera, specifically to those carrying anti-GPIIb/IIIa antibodies (48). It was found that Fc-independent desialylation is most prevalent with antibodies that target platelet glycoproteins GPIb/IX rather than GPIIb/IIIa ((50). The effects of antibodies against platelet glycoproteins on platelet destruction mechanisms have been linked to desialylation. Platelet apoptosis was found to be triggered by anti-GPIb/IX antibodies, which cause Neu1 surface translocation (48, 50).

Although in the blood bank, platelets can be stored for longer periods of time when they are refrigerated, this process can alter their function by causing factors such as the translocation of Neu1 (19). At room temperature, β-galactosidase and Neu1 exist in vesicles inside platelets; however, they become visible and active on the surface of the platelet after refrigeration. Specifically, desialylation is initiated by translocated Neu1. The aforementioned procedure primes the von Willebrand factor receptor (VWFR) complex, specifically GPIbα and glycoprotein V (GPV), to be subjected to proteolytic cleavage by metalloproteinases (MPs), with ADAM17 taking the lead. Consequently, these receptors are shed from the platelet surface. As refrigeration induces desialylation and clustering of GPIb on the platelet surface, which results in rapid clearance of re-transfused platelets *in vivo*, room temperature has become the standard for platelet storage (51, 52). Improving platelet survival rate after refrigeration is proposed by using sialidase inhibitor DANA during platelet storage (19).

Not only does GPIb’s binding to VWF result in the translocation of Neu1, but also the presence of fibrinogen, which is an important factor for blood clotting (44). After GPIb clustering, fibrinogen binds to Iib3-integrin, a fibrinogen receptor. Blocking this interaction with the RGDS peptide (Arg-Gly-Asp-Ser) reduced Neu1 translocation, indicating an Iib3-integrin-binding dependent Neu1 translocation. The same effect was observed with the sialidase inhibitor DANA, which prevents fibrinogen binding and the activation of Iib3 integrin (44).

Type 2B von Willebrand disease (vWD) is caused by mutations in the von Willebrand factor gene, which causes significant interaction between von Willebrand Factor and platelets, thus increasing aggregation and leading to thrombocytopenia (47). Glycoproteins (Iib and III) were confirmed as the specific targets of desialylation on the platelet surface (47). Indeed, the removal of Sias can result in a conformational change in integrin Iib3, exposing binding sites that allow platelets to bind to fibrinogen and other molecules, resulting in aggregation. In acquired Glanzmann thrombasthenia, which occurs in those with bleeding complications even though they do not have thrombocytopenia, autoantibodies target the Iib3 integrin receptor, causing Neu1 translocation to the platelet cell surface (53). IgG antibodies can cause platelet activation and complications if they bind to platelet receptors such as Iib3, leading to increased Neu1 translocation (53). In addition to autoantibodies against platelets, in patients with ITP and CD8+ T cells, translocation of Neu1 to the platelets’ surface increases significantly, leading to platelet clearance by phagocytosis (54). Increased desialylated platelet clearance from people with ITP has been linked to the presence of ASGPRs on the surface of hepatocytes, a class of lectin that recognizes galactose exposure on the platelets and marks them for clearance (54). However, platelet survival was significantly increased by administering pan sialidase inhibitor DANA or an ASGPRs competitor (54).

*Daboia siamensis* and Agkistrodon halys snake venom were reported to cause thrombocytopenia in humans (55). A similar mechanism was hypothesized that desialylation exposed β-galactose and β-*N*-acetyl-D-glucosamine (β-GlcNAc) in the platelets, which is recognized and engulfed by macrophages, leading to platelet clearance (55). Therefore, the neuraminidase inhibitor Oseltamivir showed potential to speed up platelet recovery by blocking sialidase activity, and thus subsequent clearance by the liver (55). However, there is no data showed whether Neu1 translocation contributes to platelet desialylation in this study.

Overall, the translocation of Neu1 to the platelet surface is critical for platelet activation, aggregation, and response to stimuli in several physiological and pathological conditions and including storage (Table 2). Therefore, Neu1 can serve as a therapeutic target in the management of many disorders that are linked to platelet dysfunction and clearance.

#### Neu1 translocation in epithelial cells

The translocation of Neu1 to the surface of epithelial cells has been confirmed. This process has significant implications for diseases and pathology, as it impacts the integrity and function of numerous body organs and tissues, including the lungs (Table 3). For instance, through its interactions with TGF-1 and other factors, Neu1 contributes to the potentiation of fibrosis in A549 and human small airway epithelial cells (56). It has been observed that human and mouse pulmonary fibrosis are often with elevated levels of sialidases, which lead to the accumulation of more pro-fibrotic cytokine transforming growth factor beta-1 (TGFβ-1) extracellularly in peripheral blood mononuclear cells, human lung epithelial cells and fibroblasts. Additionally, sialidase activity contributes to fibrosis by desialylating serum Amyloid P. Considering that the interaction between serum Amyloid P and collagen may contribute to the stabilization and persistence of amyloid deposits, desialylating may inhibit its protective activity (56).

The human insulin receptor (IR) is densely glycosylated with a variety of complex *N*-linked glycans (57). These glycans are of paramount importance in facilitating the appropriate maturation, folding, targeting, and functionality of the receptor. Therefore, glycosylation of IR is critical for the efficient transduction of insulin signals. In fact, IR that is abnormally glycosylated is incapable of dimer formation and insulin-sensitive autophosphorylation. In contrast, receptors devoid of certain glycan chains maintain consistent tyrosine kinase activity (57). It was found that Neu1 induces the formation of an IR active dimer by desialylating, leading to insulin signaling activation (58). Specifically, Neu1 removes Sia residues from IR and induces a conformational change that improves the interaction between IR subunits and activates the receptor to function properly. A study that utilized ambroxol, which is used as a treatment for lysosomal storage disease, found that it induces Neu1 expression, leading to IR activation and improves insulin signaling and glucose metabolism in HepG2 cell line, making it a promising candidate to reverse insulin resistance in Type 2 diabetes mellitus (T2DM) (58).

Membrane-spanning sialoglycoprotein mucin-1 (MUC1) is a substantial transmembrane protein that is extensively *O*-glycosylated and is typically found on epithelial cells and serves barrier and lubrication activities. MUC1 stimulates cell signaling and gene expression *via* cellular receptors, thereby facilitating cell motility, proliferation, differentiation, and survival (59). Epidermal growth factor receptor (EGFR) is a receptor tyrosine kinase that regulates survival, migration, and proliferation, among other cellular activities. EGFR communicates *via* the MAPK, PI3K, JAK, and PLCγ signaling pathways. Both MUC1 and EGFR play crucial roles in cell growth and proliferation and Neu1 can modulate their activity by desialylation and altering their affinity for their ligands, which modulates the receptor’s activity (60, 61). A study showed that Neu1 binds to the extracellular domain (ED) of MUC1 that is considered a Neu1 substrate, leading to the desialylation of MUC1-ED (60). Neu1 increases MUC1-dependent adhesion, resulting in stimulation of ERK1/2 activation. According to the findings of another study, Neu1 interacts with not only MUC1 but also the EGFR receptor (61). Actually, Neu1 inhibits EGF-stimulated EGFR autophosphorylation, a crucial step in EGFR activation. Overall, Neu1 translocation to epithelial cell surfaces can have important implications for disorders, as it participates in fibrosis enhancement, insulin signaling activation, and modulation of cell surface receptors (MUC1 and EGFR), influencing cell growth and proliferation (Table 3). Therefore, modulating Neu1 translocation to epithelial cell surfaces can be a potential therapeutic strategy for diseases like pulmonary fibrosis and diabetes.

#### Neu1 translocation in endothelial cells (ECs)

Vascular endothelial surfaces express a high level of Sias, which contribute to EC monolayer integrity, permeability, receptor activation and cellular communications as well. Desialylation of the EC monolayer impacts both its integrity and permeability. In addition, desialylation of the surface of ECs has a crucial impact on angiogenesis, endothelial adhesion to tumor cells, and leukocyte extravasation (Table 4). Neu1 has an important function in controlling sialylation on the endothelial cell surface. This is evidenced by its expression and translocation to the plasma membrane of human lung microvascular endothelial cells (HMVECs) (62). It was found that Neu1 expression on the surface of ECs causes desialylation of CD31 and disrupts CD31-driven capillary-like tube formation in HMVECs (62). CD31 (PECAM-1) is a cellular adhesion and signaling receptor abundantly present at the intercellular junctions of ECs. It serves as a regulator of leukocyte trafficking and helps preserve the junctional integrity of ECs (63). Neu1 inhibits the development of capillary-like tubes in ECs. Desialylation of CD31 by Neu1 has also been identified as a mechanism by which angiogenesis is impaired (64). It was concluded that Neu1 translocation negatively affects adhesion and migration of ECs and tube formation in ECs. Therefore, sialidase inhibitors can have an effect on the adhesion, migration, and even angiogenesis of ECs (64).

Viral infection impacts the endothelium glycocalyx (EGL), a glycoprotein and proteoglycan layers found on endothelial cell surfaces. A study demonstrated that the dengue virus non-structural protein DENV2 stimulates the translocation of Neu1 to the surface of ECs by activating TLR4 (65). After 1 to 12 h post-treatment with DENV1-4 NS1, there was a significant rise in Neu1 expression and translocation in the HPMEC cell line. Accordingly, EC monolayer integrity was compromised, and permeability was enhanced as a result of cell surface desialylation. Sialidase inhibitor Zanamivir and DANA protected these monolayers from DENV NS1-induced hyperpermeability (65). This suggests that Neu1 regulates the migratory response of ECs, which is essential for wound healing and angiogenesis. Overall, the translocation of Neu1 plays an important role in affecting the integrity, permeability, angiogenesis, and cellular connections of ECs, contributing to endothelial dysfunction (Table 4). Endothelial cell surface Sias and Neu1 expression levels are very important for EC monolayer integrity, permeability, and functions.

#### Neu1 translocation in other cells

Neu1 translocation also occurs on cells other than immune cells, platelets, epithelial cells, and endothelial cells (Table 5). Studies with aortic smooth muscle cells (AoSMCs) found that Neu1, which is likely surface localized as a component of the elastin receptor, influences elastin fiber deposition and, in turn, cellular proliferation rates (66). Elastin is a protein that gives tissues like blood vessels and skin their elasticity, therefore, elastin fibers and their proper deposition are necessary for tissue integrity, and disruptions in this process can influence cell behavior, such as proliferation rates (66). The desialylation of elastin receptors by Neu1 can alter their binding affinity for elastin, which leads to degradation, resulting in a decrease in blood vessel elasticity (66). It was found that PPCA, Neu1, and elastin-binding proteins combine to create a molecular complex that is directed to the cell surface. A study on human airway smooth muscle cells (ASMCs) suggested that Neu1 plays a role in the formation and organization of elastic fibers in tissues (35). Modulation of Neu1 activity by sialidase inhibitor DANA and Zanamivir impacts elastogenesis, which is significant in the regulation of elastic fiber formation (27).

Neu1 is found at the plasma membrane of COS-7 cells with both the C-terminus and N-terminus of Neu1 in the cytosol, indicating a type 2 (TM2) membrane protein (24). Surprisingly, PPCA was not detected in the biotinylated part, which means it does not translocate with Neu1 to the plasma membrane. This indicates that Neu1 and PPCA do not form a complex at the plasma membrane (24). However, the same study confirmed that macrophages have cell surface expression of both Neu1 and PPCA. Further investigation was focused on the predicted transmembrane segment Neu1/TM2, which was comprised of residues 316 to 333. Point mutations in the transmembrane domain disrupt Neu1 dimerization and reduce sialidase activity, which highlight the functional relevance of Neu1 dimer formation (24).

The interaction between the elastin receptor complex and elastin degradation products (EDPs), specifically kE, is the mechanism by which Neu1 is translocated to the insulin receptor in 3T3-L1 adipocytes (67). The translocation of Neu1 to the membrane increases significantly with EDPs treatment, where it interacts with the β-chain of IR and affect its sialylation level (67). Furthermore, an animal model was utilized to demonstrate that the intravenous administration of kE to fasting mice resulted in a significant increase in blood glucose levels, indicating the existence of a hyperglycemic effect. This effect was dosage-dependent and insulin-independent in the liver, white adipose tissue, and soleus muscle, among other tissues. They confirmed these results by using coimmunoprecipitation experiments, which concluded a strong interaction between Neu1 and IR treated with EDPs (67). Therefore, inhibition of Neu1 activity and EDP binding to elastin-binding protein can reduce insulin resistance caused by EDPs. Another investigation with the above-mentioned cell line, 3T3-L1 adipocytes, confirmed the concept that Neu1 modulates the promoter activity of IL-6 and MCP-1, thereby indicating a regulatory function in their expression. The inhibition of Neu1 leads to a decrease in the nuclear localization of NF-κB p50 and p65, which are transcription factors that are crucial for inflammation. Significantly, sialylation of TLR4 was elevated in adipocytes activated with LPS when Neu1 was knocked down. This finding suggests that Neu1 is involved in the TLR4/NF-κB signaling pathway (68).

An earlier study found that neurotrophin-induced Trk tyrosine kinase receptor activation and neuronal cell survival responses are under the control of Neu1 sialidase (39). They found that nerve NGF binding to TrkA induces Neu1 sialidase activation in live primary neurons and TrkA- and TrkB-expressing cell lines. They confirmed the Neu1 localization by flow cytometry and confocal microscopy, which showed a significant increase on the cell surface. In addition, the activation of MMP-9, which is facilitated by Trk activation after neurotrophin binding to the receptor, enhances G-protein coupled receptor (GPCR)-signaling, as well as the activity of Neu1 sialidase on the cell surface. Also, Tamiflu completely blocks Neu1 sialidase activity in live TrkA-PC12 cells treated with NGF and subsequent inhibition of Trk activation in primary neurons and neurite outgrowth in TrkA-PC12 cells. These findings confirmed that cell surface translocation of Neu1 and MMP-9 crosstalk on the cell surface are critically essential for neurotrophin-induced Trk tyrosine kinase receptor activation and cellular signaling (39).

Overall, Neu1 translocation in different cells plays a crucial role in a wide variety of biological processes, including the deposition of elastin fibers, the rates of cellular proliferation, the inhibition of insulin signaling, and the development of aortic dissection (Table 5). Therefore, Neu1 is important in maintaining tissue integrity and cellular behavior, and a variety of pathological conditions are highlighted by the fact that it interacts with and is distributed across different cell types.

### Neu2 sialidase translocation

Neu2 is typically present in the cytosol, but its presence at the cell membrane is crucial for some pathological and physiological conditions. It was found that platelet activation and adhesion stimulate the translocation of Neu2 to the cell surface (44, 69). In particular, levels of membrane associated Neu2 levels increase significantly in response to Ristocetin stimulation. In the presence of von Willebrand factor and GPIb also known as the CD42 complex, Neu2 on the platelet surface modulates the glycosylation of GPIb and, consequently, prevents platelet agglutination in the blood (44). Moreover, Neu2 activity is involved in fibrinogen binding to αIIbβ3-integrin, an essential step in platelet aggregation, a critical mechanism in the development of blood clots (44). In cancer cells, overexpressed Neu2 was able to translocate to the cell membrane and desialylate and activate Fas by removing α2,6-linked Sia, leading to induction of the extrinsic apoptotic pathway (69). Activated Fas catalyzes the phosphorylation of a series of proteins leading to apoptosis. Through co-immunoprecipitation assays, they confirmed the existence of Neu2 on the cell surface and its connection with Fas desialylation (69).

### Neu3 sialidase translocation

Neu3 is associated with the plasma membrane, where it desialylates membrane gangliosides in various biological processes (23). A study suggested that Neu3 functions as an S-acylated transmembrane protein, wherein the C-terminus is exposed to the cytosol and the remaining segment extends into the extracellular environment (70). The localization of Neu3 changes in response to different stimuli, *e.g.*, activation and differentiation of immune cells (71, 72, 73). One study found that Olanzapine, an antipsychotic agent associated with insulin resistance, induced Neu3 activity on the cell surface, which surprisingly inhibited Neu1 activity. This interferes the Neu1-MMP9-GPCR signaling pathway, leading to a subsequent disruption of the insulin receptor function. As a result, this contributed to the attenuation of insulin-induced IGF-R and IRS1 phosphorylation, leading to insulin resistance (74). The Neu3 inhibitor DANA and anti-Neu3 neutralizing antibodies were utilized in this study to confirm the association between Neu3 and olanzapine treatment (74). Also, Neu3 translocation in response to stimuli is demonstrated by the fact that phosphatidic acid (PA) stimulates the activity of Neu3 and increases Neu3 activity by 4 to 5 times in response to a concentration of 50 mM of PA. Neu3 is involved in regulating the structure of gangliosides which is crucial for many cells signaling pathways, including Ras-signaling which facilitates cell migration (71).

Neu3 is also associated with the endocytic/recycling pathway and functions at the endosomal membrane (72, 73). One study demonstrated that Neu3 is a peripheral membrane protein (75). In response to appropriate stimuli, endosomal Neu3 is shifted to the plasma membrane (76). GRP78 modulates Neu3 folding and transport from endosomes to the cell membrane. The rate of this process is proportional to the concentration of glucose. Using a GRP78 inhibitor, the same study verified Neu3 translocation (76). In addition, Neu3 rapidly mobilizes to the ruffle cell membranes in squamous carcinoma A431 cells in the presence of EGF (epidermal growth factor) (77). Neu3 co-localizes with the Rac-1, a protein involved in cellular signaling and cytoskeleton rearrangement, during this key cellular process (77). Further, a significantly increased translocation of Neu3 to the plasma membrane occurs under hypoxic conditions, causing the decrease of GM3 and activation of the hypoxia-inducible factor (HIF)-1 and the epidermal growth factor receptor (EGFR) signaling pathway, which inhibits programmed cell death and enhances cellular resistance (78). Therefore, Neu3 translocation is involved in various biological pathways and may serve as a modulation target accordingly.

### Neu4 sialidase translocation

There are two forms of Neu4, differing in the first 12 N-terminal amino acids (79, 80). The long form was in lysosomes (13) and mitochondria (14, 80), whereas the short isoform is associated with ER membranes (79, 80). In addition to lysosomes, mitochondria, and ER, Neu4 can translocate to the cell surface (11, 13, 14). One study suggested that Neu4 is associated with the cell membrane of platelets following VWF and Ristocetin treatment, which was also confirmed by platelet apheresis resulting from shear force (44). It was demonstrated that Neu4 is released from its intracellular stores upon GPIbα-clustering (81). As a result of hypoxia, the localization and activity of Neu4 may be changed, resulting in its accumulation at the plasma membranes of DLD-1 colon cancer cells (82). In addition, Neu4 staining was detected on the cell surface of Ra2 microglia both before and after LPS stimulation (37). Another study found that thymoquinone (TQ) induced Neu4 translocation in a variety of cell types, including naive primary macrophage cells and type I Sialidosis fibroblast cells (83). This translocation occurred *via* G-protein-coupled receptor (GPCR) signaling, which is sufficient to activate MMP-9 (83). Additionally, it was found that intense Neu4 activity appeared in the cell membrane of hippocampal CA3 pyramidal cells following neural excitation (84). Overall, Neu4 can be involved in cell death or brain development through a change in its subcellular localization (84).

### Sialidase translocation mechanisms

Mammalian sialidases play unique roles in different subcellular localizations *via* their intracellular shifts upon the change in physiological and pathological conditions. Despite various hypothesized processes, the detailed molecular mechanisms for sialidase translocation remain unclear. An earlier study by Lukong *et al.* suggested that plasma membrane associated Neu1 may be retained at the surface by Tyr (412) phosphorylation of the C- terminal YGTL-signal peptide (22). This peptide sequence is known as a plasma membrane to endosome targeting motif and its phosphorylation supposedly blocks clathrin-mediated endocytosis (22). This, however, implies that the C-terminus is accessible to cytosolic kinases and that Neu1 is an integral membrane protein. Another study suggested a lysosomal exocytosis mechanism, in which Neu1 together with PPCA translocate to the cell surface of macrophages in vesicles budding off the lysosomes and fusing with the plasma membrane (40). While others suggest that Neu1 translocates to the plasma membrane by endocytic vesicles. The link between Neu1 and lipid rafts in cells was shown in one study to be crucial for the translocation across the plasma membrane (85). To establish this correlation, they utilized methyl-β-cyclodextrin (MCD), a substance that effectively depletes lipid rafts and sequesters cholesterol, which inhibits the translocation of Neu1 to the cell membrane (85). Phosphorylation processes, lipid rafts, transport through vesicles, the participation of endocytic vesicles, and the possible involvement of particular protein domains all play important roles in the translocation of Neu1 to the cell surface. So far, there have been no mechanism studies on Neu2 and Neu4 sialidase reported yet. Similar to lysosomal Neu1, the lysosomal exocytosis mechanism can be applied to Neu4 in the lysosome. The molecular mechanisms of all four sialidases’ translocation deserve full investigation, which will contribute to a better understanding of sialidases’ function and related physiological and pathological pathways.

## Secretion of sialidases

**Figure 3:**
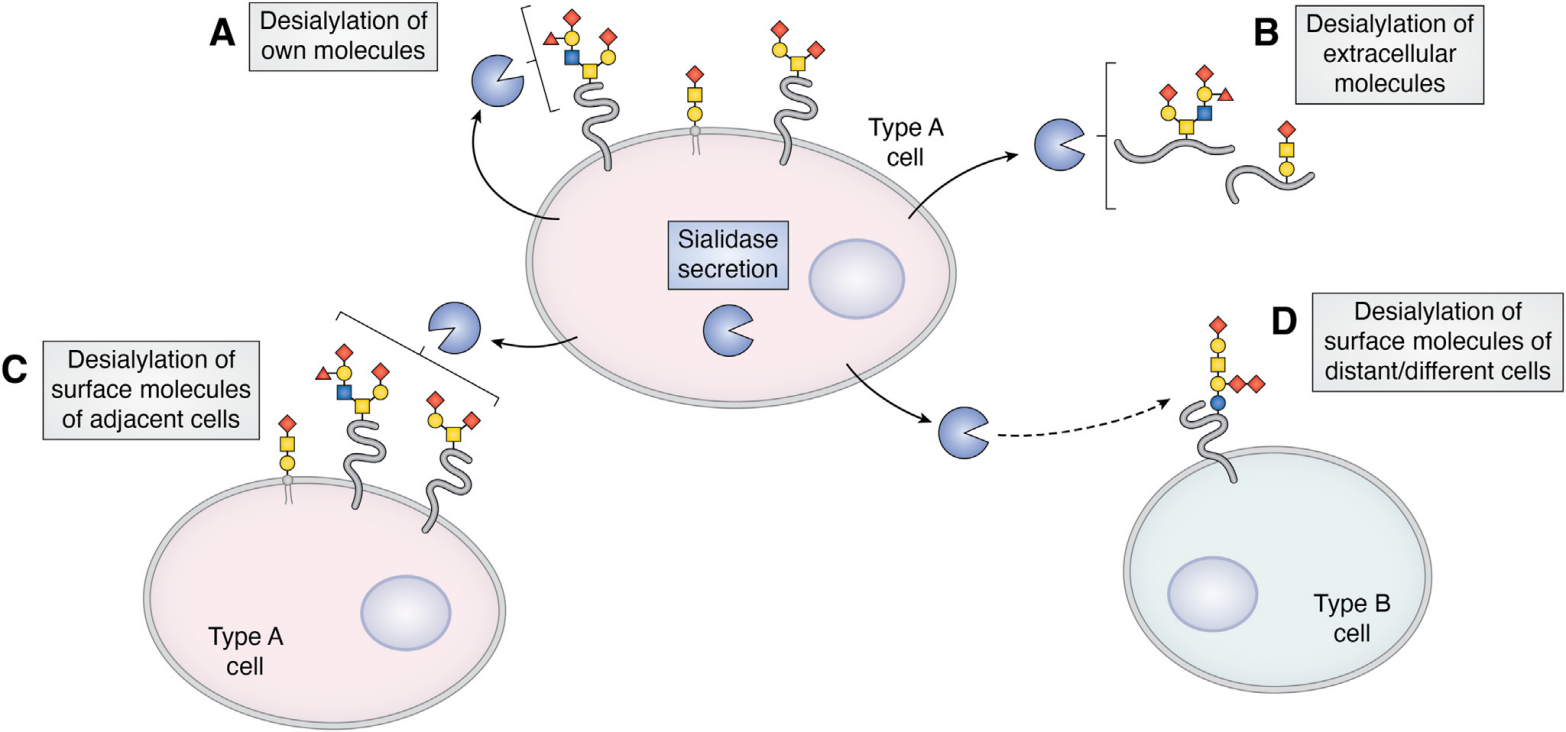
**The cell secretes sialidase and secretedsialidase serves as an extracellular sialidase.** Secreted sialidase causes the desialylation of cell surface glycoprotein and glycolipid of its own (*A*), extracellular glycoprotein and glycolipid (*B*), and cell surface glycoprotein and glycolipid of adjacent same (*C*) and different (*D*) cell.

Sialidases are intracellular enzymes, but their activities are detected in the extracellular space of various cells including fibroblasts, CHO cells and activated microglia cells, indicating the secretion of sialidases from the cells. This secretion could either be attributed to pathological conditions or be a result of normal physiological processes. Mammalian cells may utilize the traditional secretory pathway to secrete sialidases; this pathway includes the synthesis of the enzyme in the endoplasmic reticulum, further processing in the Golgi apparatus, and then secretion (86). Many studies have confirmed that sialidases can be secreted into the extracellular space from the cells upon stimulation. Secreted sialidases could serve as extracellular sialidases, which cause the desialylation of both extracellular glycoproteins and glycolipids and cell surface glycoproteins and glycolipids of their own and adjacent cells and participating in various biological pathways (Fig. 3). This section discusses the secretion of sialidases that had been reported so far, related to their activity, different forms, and secretion mechanisms.

### Extracellular sialidases

In 1988, Usuki *et al.* first reported the sialidase activity in the culture medium of human skin fibroblasts (87). They found that two or more forms of sialidase are accumulated in the medium and proposed that human fibroblasts release sialidase activity into the medium during growth. Later the same group reported the "neutral form" of sialidase activity in the conditioned medium of cultured human fibroblasts (88). Chinese Hamster Ovary (CHO) cells are the most common mammalian cell line used to express recombinant glycoproteins with human-like glycosylation modifications for therapeutic applications (89). Maintaining the Sia content of recombinant glycoproteins, particularly those intended as therapeutics, is essential since Sia affects the physical, chemical and immunogenic properties of the glycoprotein molecule (90). Several studies have detected stable sialidase activity in the culture supernatant of CHO cell lines (21). Soluble sialidase that degrades recombinant glycoproteins expressed in CHO cells was isolated and purified (91). It is now known that cytosolic sialidase derived from CHO cells retained a significant amount of activity in culture supernatant (21, 92), and it is specific to α 2,3-linked terminal Sia (8), which is the main linkage produced in CHO-K1 cells (93). Therefore, it is a great concern that glycoproteins produced by CHO cells are prone to losing terminal Sias. A detailed understanding of the extracellular Sia degradation of recombinant glycoproteins in the culture environment of the CHO bioprocess is in high demand.

Sialidase activities were also detected in the cell culture medium of the LPS activated microglia cells (94, 95), indicating the secretion of sialidases from the cells upon activation. Interestingly, in the cell culture medium sialidase activities are high enough to cause the desialylation of PC12 (neuron-like) cells (29). In addition, activated BV-2 murine microglia secrete extracellular vesicles (EVs) containing Neu3 that can desialylate neurons and disrupt their normal communication (96). This indicates that secreted Neu3 may be a link between neuroinflammation and alterations in neuronal electrophysiology (96). Another study also provided evidence of sialidase being released into the extracellular environment when preserved platelets were re-warmed (19). Warming the platelets after storage results in the secretion of Neu1 and Neu3, which are responsible for removing Sias from the platelet von Willebrand factor receptor (VWFR) and targeting the GPIb subunit (19). All these results indicate that secreted sialidases are involved in various biological pathways.

### Exosomal sialidases

Exosomes are a subset of EVs originating from the endosomal system and released in the extracellular milieu. Exosomes are pivotal in intercellular communication and are associated with a multitude of pathophysiological pathways, such as immune response and inflammation (97). Neu1 was confirmed to be localized on exosomes (37, 98). One study found that a microglial cell line (Ra-2) releases Neu1 in exosomes when stimulated with LPS (37). Neu1 appeared to localize the membrane of these vesicles, as indicated by the fact that isolated and intact exosomes showed sialidase activity. In addition, Neu3 secreted from the HeLa cell line is associated with exosomes, indicating that it is present in the culture medium as exosomal sialidases (75). By using the retinal pigment epithelium (RPE) cell as a model, sialidase is present on the surface of EVs from oxidatively stressed donor cells, according to a study examining the mechanisms of EV uptake by recipient cells (stress EVs) (99). They suggested that EV uptake involving sialidase-containing EVs and the mechanisms employed by viruses for cell entry are identical since both use sialidase for entry. Once sialidase has been localized to the exosome surface, it can interact with molecules in the cytoplasm, the plasma membrane, and the soluble and insoluble components of the extracellular matrix. Therefore, exosomal sialidases play important roles in both physiological and pathological pathways.

### Sialidase secretion mechanisms

As described above, cells secrete sialidases during cell growth and upon stimulation and several forms of sialidases have been confirmed, including soluble and exosomal sialidases. Although the molecular mechanisms for sialidase secretion are still unclear, three mechanisms can be proposed from the cells: lysosomal exocytosis, shedding and exosome secretion. Soluble sialidases in the extracellular compartment may be derived from one of the transport pathways for the secretion of lysosomal enzymes. It has long been known that cultured fibroblasts secrete lysosomal enzymes. It would not be surprising to detect lysosomal sialidase activity in the conditioned medium of fibroblasts, derived from this pathway of secretion. In addition, sialidases in the medium can be the products of proteolytic action at the extracellular surface, in which case they should have sequence homology with regions of a plasma membrane-associated sialidase. In addition, sialidases can be released into the medium bound to exosomes.

#### Lysosomal exocytosis pathway

Lysosomal exocytosis is a potential pathway involved in the secretion of Neu1. Using an inhibitor of lysosomal exocytosis, vacuolin-1, Allendorf *et al.* (29) observed a reduction in extracellular sialidase activity from both BV-2 cells and primary rat microglia, indicating that lysosomal exocytosis is involved in the secretion of sialidase activity. Lysosomal exocytosis is calcium-dependent. Calcium ions are involved in the regulation of lysosome fusion with the plasma membrane. An increase in calcium concentration facilitates the fusion process. Intracellular calcium chelator BAPTA-AM inhibits lysosomal exocytosis and blocks LPS-induced release of extracellular sialidase in BV-2 and primary rat microglia (100). Intracellular esterases break the AM groups once BAPTA-AM enters the cell, turning it into its active form, supporting the role of lysosomal exocytosis in sialidase release. Nevertheless, a well-characterized inhibitor of exosome formation and release, GW4869, did not prevent sialidase release from BV-2 microglia (100).

#### Sialidase shedding

Ectodomain shedding is a proteolytic process that regulates the functionality and abundance of numerous membrane proteins, including sialidases (101). Neu1 is primarily located in lysosomes and can be translated to cell surfaces. Neu3 is on the plasma membranes. Both of them could undergo proteolytic release from the membrane. An earlier study by Ogura *et al.* indicated sialidase release by proteolytic cleavage of the plasma membrane-bound sialidase (101). In their study, a ganglioside sialidase assay was utilized for an evaluation of the chromatographic properties of the conditioned medium collected from preconfluent cultures (101). Specifically, the concentrate of the medium was applied to a gel filtration column of Sephacryl S-200 HR. Two sialidase activity peaks in the elution profile occurred at pH 6.5, which had estimated molecular weights of 16 and 47 to 69 kDa, respectively. The sialidase activity of the higher molecular weight form was increased about 6-fold by the addition of Triton CF-54, whereas the 16-kDa form did not require detergent (88). Sialidase released to the medium *via* proteolytic cleavage is especially attractive in view of the low molecular mass (16 kDa) of one of the ganglioside sialidases reported. A comparison of the primary sequences of the 16 kDa sialidase in the medium and the plasma membrane sialidase will be necessary to evaluate the possible shedding mechanism. Further characterization of purified protein will be needed to clarify this possibility as well.

#### Exosome secretion pathway

Exosomes are nanosized membrane vesicles, and their biogenesis and secretion depend on complicated biological processes, including a cytoplasmic multi-subunit system essential for membrane remodeling, vesicle budding and cargo sorting in endosomes or multivesicular bodies. Recent studies have demonstrated that exosomes contain higher amounts of enzymes in response to different cellular stimulations (102, 103). Neu1 is typically found in the lysosome, along the lysosomal membrane and lumen (10). The main function of Neu1 is to degrade internalized or endocytosed sialylglycans (7). Therefore, Neu1 can be involved in exosome biogenesis and secretion. A study conducted with the microglial cell line Ra-2 found that Neu1 was released into the medium bound to exosomes (37). In addition, Neu3 was released from the HeLa cell line into the culture medium as exosomal sialidases (75). Therefore, sialidases can be released through the exosome secretion pathway.

Overall, there have been no comprehensive functional or mechanistic investigations into the secretion of sialidases from cells. Furthermore, the physiological function of the secreted sialidases continues to be the subject of deeper research. This includes their involvement in the modification of cell surface receptors and their activation. This is especially important regarding each individual Neu1, Neu2, Neu3 and Neu4 as each of them has different biological functions and is involved in different physiological and pathological pathways.

### Biological effects of extracellular sialidases

The accumulation of sialidases in the extracellular fluid could be expected to exert a biological effect. An early study conducted in the 1990s established that extracellular sialidase is secreted into the cell culture supernatant due to cellular membrane disruption. This enzyme was found to be responsible for the desialylation of recombinant glycoproteins generated by CHO cells (91, 92, 104). A 2015 report demonstrated that the extracellular sialidase could degrade polySia on Ra2 cells in a *cis* mode but also on other cells in a *trans* mode (37). A 2017 study indicated that the sialidase activity secreted by activated microglia was sufficient to desialylate PC12 cells and that the level of desialylation is comparable to that induced by added sialidase (95). The most recent study confirmed that sialidase released by microglia could desialylate neurons, thereby changing neuronal activities (29). Another recent report showed that incubation of human neutrophils with the extracellular human sialidase Neu3, but not Neu1, Neu2 or Neu4, induces morphological change of neutrophils from a round to a more amoeboid. This causes the primed human neutrophil markers CD11b, CD18, and CD66a to localize to the cell cortex. However, it decreases the localization of the unprimed human neutrophil markers CD43 and CD62-L at the cell cortex (105). These results indicate that extracellular Neu3 can prime human neutrophils and serve as a potential regulator of inflammation.

Overall, earlier studies in the 1980s confirmed the soluble sialidases accumulating in the medium of cultured fibroblasts. Now, it is known that the release of sialidases from various cell types but the origin of these sialidases remains to be determined. It would seem reasonable to expect that they are at least partially derived from a vesicle-mediated transport system from Golgi apparatus and trans-Golgi network and that these forms are likely to be lysosomal in nature. It may be that some of the sialidases in the medium are products of proteolytic action at the extracellular surface, in which case they should have sequence homology to portions of a plasma membrane sialidase. Finally, it is plausible that there are completely novel secreted sialidases which are exported to carry out specific cell surface-mediated functional roles. More intensive research is needed to distinguish between these possibilities and also to determine further if and how extracellular sialidases modulate signaling events.

## Summary and future perspective

Sialidases are widely distributed in nature and are involved in many physiological and pathological processes. In mammalian cells, there are four genetically distinct sialidases (Neu1, Neu2, Neu3, and Neu4). Their cellular locations and substrates are different in the cells. Furthermore, the expression levels of Neu1-4 can vary in cells under pathogen stimulation. They could translocate to a specific subcellular location upon cell activation, where they could cause desialylation of a specific biomolecule and contribute to physiological and pathological pathways as well (Fig. 2). However, the extent to which the majority of mechanisms regulating different sialidases are associated with the localization of sialidase activity is still unknown. The use of a site-specific probe to perform precise and sensitive *in situ* sialidase imaging would be advantageous in the examination of sialidase activity within cells and tissues, potentially revealing novel sialidase functions.

Transmembrane (TM) domains have an essential function in determining the location and activity of sialidases on the cell surface. This is because they regulate the interaction with sialidases and their substrates and regulatory proteins. It was thought that Neu1 had two possible transmembrane (TM) helical sections, TM1 (residues 139–159) and TM2 (residues 316–333), which were believed to help in translocation to the cell membrane (106, 107). Recent structural study indicate that these regions are β strands located in the protein core, rather than functioning as transmembrane (TM) domains. This suggests that Neu1 connects with membranes by interactions with other proteins or processes (35, 40, 60, 108). In this review, we discussed several potential proteins which help in the translocation of Neu1. These proteins include TLR4, CD36, CD11b/CD18 (β2-integrin), TrkA receptors, protective protein/cathepsin A (PPCA), glycoprotein Ib (GPIb), epidermal growth factor receptor (EGFR), CD31, elastin receptor, insulin receptor (IR), and nerve growth factor (NGF).

Although Neu2 lacks TM domains and is known as a soluble protein, it could still have an indirect interaction with the membrane through interactions with membrane-bound proteins or receptors (44, 76, 109). The mechanism of Neu3 interaction with the membrane is a subject of debate, similar to Neu1. The initial predictions indicated that Neu3 contains a transmembrane helix (18, 109, 110). More recently, Neu3 has been classified as an outer membrane protein, rather than a core membrane protein with a typical transmembrane domain. This indicates that Neu3 is linked with the plasma membrane's outer leaflet but does not span the membrane with a transmembrane helix (70, 111). Although Neu3 is mostly located on the plasma membrane, it is also present in endosomes. Some studies hypothesize that it can translocate to the cell surface by endocytosis (76, 107). Many studies confirmed that protein-protein interactions facilitate Neu4 translocation to the plasma membrane, instead of to direct membrane anchoring (14). Even though some suggests the presence of two strong transmembrane helices in the central region of Neu4 (79, 110). In general, the various mechanisms of membrane association among the sialidases emphasize the complex nature of their regulation and function in cellular environments.

Understanding how glycan modifications affect immune cell function as a result of Neu1 translocation could be aided by more investigation into these changes and their consequences. Also, Neu1 translocation is closely associated with platelet-related disorders. Targeted therapies for platelet-related disorders can be developed by elucidating the mechanisms that control Neu1 translocation to the cell surface. Diseases linked to abnormal aggregation and activation of platelets, such as immune thrombocytopenia (ITP) and von Willebrand disease (vWD), could be affected by regulating Neu1's membrane activity. The discovery that Neu1 plays a role in the activation of numerous receptors makes it an extremely useful target for the management of type 2 diabetes (T2DM), fibrosis, and elastic fiber regulation. The functional consequences of Neu1 translocation in cancer progression and metastasis are worth investigating because they have been observed in a number of different cancer cell lines. Therefore, developing inhibitors, antibodies, or gene therapies that selectively target Neu1 may open the path for better treatment of many disorders and diseases.

On the other hand, the cells secrete sialidases upon activation, which serve as extracellular sialidases. The secreted sialidases cause the desialylation of both extracellular glycoproteins and glycolipids and cell surface glycoproteins and glycolipids on their own and adjacent cells and are thus often involved in pathological pathways (Fig. 3). Extracellular sialidases, including exosomal sialidases, may function as biomarkers for specific diseases. Mammalian cells, including activated microglia, fibroblasts, and CHO cells, have been recognized as sources of extracellular sialidases. Extracellular sialidases, exosomal sialidases, and sialidase shedding are the three distinctions among the extracellular sialidases.

Overall, the molecular mechanisms of sialidase translocation and secretion are still unclear. In particular, the currently available tools for interrogating the translocation and secretion of sialidases fall short, which limits the study of the role of sialidases, their translocation and secretion mechanisms related to physiological and pathological pathways. Both carbohydrate chemistry, glycobiology, chemical biology, and molecular and cellular biology approaches are highly needed to tackle this unmet need. For example, selective and location specific sialidase substrates and probes will be powerful tools for measuring and imaging specific sialidase activity and levels in a specific cellular compartment in the cells upon activation and stimulation. In addition, selective location-specific inhibitors are useful for inhibiting critical enzymes’ activity and their translocation as well. Furthermore, it is necessary to investigate both soluble and exosomal sialidase releases from the cells upon different stimuli. Additionally, it is important to determine how the sialidase is associated with exosomes, *i.e.*, whether it is encapsulated within exosomes or exists on their surface. In terms of therapeutics, targeted exosomes containing sialidases on their surface will be promising for the treatment of inflammation diseases and cancer. Eventually, future research is highly expected and will lead to the discovery of the molecular mechanism of sialidase translocation and secretion in controlling signaling pathways and in diseases like infection, inflammation, immune disorders, thrombocytopenia, and cancer, as well as the development of new therapeutic targets and treatments.

## Conflict of interest

The authors declare that they have no conflicts of interests with the contents of this article.
